# Milk in the Sickroom Dietary1Read at the thirty-first annual meeting of the Medical Association of Central New York, at Auburn, October 18, 1898.

**Published:** 1898-12

**Authors:** B. C. Loveland

**Affiliations:** Clifton Springs, N. Y.


### BUFFALO MEDICAL JOURNAL.

VOL. XXXVIII. DECEMBER, 1898. No. 5.

### Original Communications.

### MILK IN THE SICKROOM DIETARY.[1]

BY B. C. LOVELAND, M. D., Clifton Springs, N. Y.

THAT the value of milk as a therapeutic prescription is almost unknown by the laity is in accordance with the observation of all medical men. That the extent and variety of uses to which it may be put in combating disease is also unknown by many practitioners of medicine is the conviction arrived at after several years of study and experience. Hence I have chosen this subject.

I think that 90 or more per cent. of the patients for whom I prescribe a diet, in which milk is mentioned as an important factor, if they could be brought together, would form one magnificent chorus, and the theme which would engage all their voices would be " I never could take milk. It never did agree with me," and a few would add, " I never could even take it as a baby without its making me sick." So common is this experience and so generally do I find that these same persons meet with a new revelation, and learn that they cannot only tolerate milk, but derive great benefit from its use, that I have ventured to record some of my observations and conclusions.

Of course, if milk would only agree with our patients, we would like to give it in all kinds of diseases attended with loss of flesh and general poor nutrition. And it is equally true that milk, given as it usually is, does not agree with many of these patients. If these statements are correct and if, as our physiologists say, milk is the only perfect food for man, we must look to some error in the administration for the reason why so many of our patients are unable to take it with benefit.

1. Read at the thirty-first annual meeting of the Medical Association of Central New York, at Auburn, October 18, 1898.

The ways in which milk disagrees with patients are chiefly the following: (I will mention them in the order of their frequency) "biliousness," constipation and dizziness, acidity of the stomach, indigestion and diarrhea. Taking these up in order we will study the causes and the remedy for the disagreement in each case. "Biliousness," acute lithemia, that condition of malaise, dull heavy head, loss of appetite, with possible furred tongue and nausea, usually occurs in the so-called bilious temperament, a condition generally attended with constipation and deficient kidney and skin excretion and blood rich enough or even too rich in all the essential ingredients except water.

A dizzy head is the legitimate result of these conditions, whether the patient is taking milk or not. This condition frequently follows the use of milk as an aid to gaining flesh, the patient often enriching it by the addition of cream and refraining from the use of even the small amount of water such patients usually drink, for the milk quenches thirst and most people only drink when thirsty. The blood, already too thick, becomes thicker. The water contained in the milk, being in reality a distilled water, possesses very marked diuretic properties. On the addition of milk to the dietary in the manner just mentioned, there is an immediate and considerable increase in the quantity of urine excreted, and this increase is in a measure at the expense of the fluid usually kept in the intestinal secretions. Consequently, unless the patient is made aware of the necessity of drinking the usual amount of water in addition to the milk, we find that this concentration, of the intestinal secretions and liver secretion as well, results in constipation, biliousness, furred tongue, dizzy head and the other symptoms grouped in this class.

The remedy for this condition, or, in other words, the way to adjust milk to a patient such as has just been described, is to specifically include in the dietary such an amount of water and the coarser foods, such as the entire grain cereals and green vegetables, (most fruits being contraindicated with milk,) as will supply the necessary materials to keep the intestinal contents of a proper consistency and thus prevent the constipation and other results referred to. To be more specific, in cases where milk is given as an addition to the diet for the purpose of increasing flesh or the general nutrition, if we prescribe three glasses of milk during the twenty-four hours, our patient will require the usual nine glasses of water per day and some portion of this may well be taken with the milk. If we prescribe six glasses of milk per day, in the same manner six or seven glasses of water per day will be required to supply the necessary amount of fluid. As we still further increase milk, using less solid food, we may use less water, until we have our patient on a pure milk diet, in which circumstances at least twelve glasses or three quarts of milk in twenty-four hours will be prescribed, and our patients will scarcely need any more water than the little they may desire, which will hardly need to be ordered by the physician. But in directing a dietary in which milk forms only a portion of the daily food, the prescribing of a definite amount of water is as important as the prescribing of a definite amount of milk.

When the habit of constipation has existed for a long time, such a prescription of diet will not immediately correct it, but with the aid of other rational measures for the correction of the habit will sooner or later conquer it.

Sour or acid stomach is frequently complained of by the patient as a result of taking milk as a part of the dietary. This usually occurs in those who have a hyperacid stomach to begin with, and the acidity following immediately the taking of milk is usually simply the natural stomach acid, made apparent by the eructation which sometimes follows directly on filling the stomach with fluid. It is wise, then, to go carefully into the subject when the patient complains of acidity, to ascertain whether it is as just stated or is really indigestion. A glass or two of hot water usually is sufficiently corrective in this condition.

Indigestion, either real or imaginary, is often complained of when the use of milk is urged and nine times out of ten the patient will attribute such a feeling to the milk, for, as has already been stated, most patients expect milk to disagree with them. This feeling may be only the feeling of distension or load in the stomach immediately after taking the milk, not indigestion at all and in no wise a contraindication to the use of the milk. The stomach had not been accustomed to such a degree of distension as is now required of it and the result is a temporary feeling of fulness, often mistaken for indigestion.

When indigestion does occur, it is because of the inability of the stomach to secrete digestive ferments, such combinations of food materials as to induce fermentation, eating when over-tired or under strong emotion, in fact any of the causes that would provoke indigestion under any circumstances. I have observed one instance, curiously enough, in which the stomach fluid showed neither hydrochloric acid nor peptonising ferments in which milk was evidently digested, probably passing into the intestine first, but fermentation was easily induced and often hard to overcome.

If for any reason milk seems specially indicated, it does not do to stop on the statement by the patient that the milk disagrees or does not digest. If indigestion occurs it will generally show itself in a diarrhea containing curds or cheesy masses, or the patient will vomit curds, not simply small white flecks, which is the normal condition of milk partially digested. In this shape nervous patients will often bring up a little, and think they have abundant evidence of indigestion. I have referred to diarrhea in connection with the indigestion mentioned. It does occur but very rarely, as most milk patients suffer with constipation.

Milk, if necessary, diluted with water will digest in the stomach if anything will, and if we find indigestion, search out the cause and remove it, be it fruit, improper amount of exercise, improper mode of eating or any other source of indigestion. Leave the stomach empty of milk for twenty-four hours, use freely of water, and try it again, and it will probably agree. If it is necessary for the patient to take any food while waiting for the milk ferment to be cleared from the stomach, raw eggs or the whites of raw eggs can be used. The stomach is a receptacle as well as a digestive organ and any receptacle for milk which becomes infected with the milk ferment must be kept empty of milk till, what with the washing with water and withholding the special food for the milk germ, they are cleared out from the stomach.

One or two cases will be sufficient to illustrate :

CASE I.—Mrs. H. L. W., 57 years of age, was brought to me June 11, 1894. She had good health till four years previous, when she had suffered a nervous shock, following a wound in the ankle produced by a pair of scissors falling on the limb. In spite of good medical advice the patient suffered from continuous loss of flesh, sleeplessness, loss of appetite, chronic constipation, distress in the stomach and the like, until when this history was taken, her weight had dropped from 155 lbs., her weight in health, to 86 lbs. Physical examination was negative so far as organic disease was concerned. Skin was very flaccid and flabby, as were all her other tissues. Urine was normal. Diagnosis was chronic nervous dyspepsia (neurasthenia gastrica), with catarrhal gastritis.

*Treatment.*—Tincture nux vomica, 5 drops three times a day and phosphate of soda, 10 grains, in a glass of hot water a half hour before each meal. Diet as follows: Breakfast, chopped steak, graham gem and two glasses of milk. 10.30 A. M., two glasses of milk and a raw egg. 12.30, dinner, roast beef or lamb, one gem or one slice of graham bread and two glasses of milk. 3.30 P. M., lunch, two glasses of milk and a raw egg. 5.30 P. M., one gluten roll, one dish of cracked wheat or oatmeal and two glasses of milk. 9 P. M., two glasses of milk and a raw egg. When this régime was given her, she raised both hands in "holy horror" and said, "My goodness, doctor, I never can take milk; I never could take it when I was well. Why, in three days I will be so dizzy that I will be unable to turn my head on my pillow, to say nothing of sitting up." It is unnecessary to explain how she was induced to take the diet as recommended, but she did so with the most satisfactory results. Her dizzy head never came. Some aid was required for a time to secure evacuation of the bowels, but soon the diet righted this. She made an average gain in flesh of three pounds per week for twelve weeks, and when she left my care she weighed 147 lbs. Now, after four years, she is still well and weighs 152 lbs. and has been on a mixed diet for the past three and a half years or more.

CASE II.—Rev. H. T., brought to me July 29, 1897; aged 21 years; height, 6 feet 1 inch; weight, 119 lbs. Had a marked pigeon chest, nervous temperament; a hard student. His father was asthmatic, dyspeptic and pigeon-chested. He had always been underweight, and dyspeptic and constipated all through his college life. For two years had depended on enemata for moving his bowels. Suffered with extreme distension with gas, roaring in head, distress in back of neck and head, great weakness, also weakness of voice. Physical examination, negative, except a distended stomach. Blood, normal; urine, concentrated, but normal. Diagnosis, neurasthenia gastrica with gastrectasis.

*Treatment.*—Sodii phosphate, 10 grains, in a glass of hot water, three times a day. Diet: 2 glasses of milk and a raw egg six times a day, and also two or three times in the night, if awake. Other food being added after a week or so as he could bear it. He soon became hungry, a thing he had not known for months, and increased the milk beyond my prescription. Too many details would be wearisome, so that I will simply state that the result was an average gain of 5 lbs. per week for three months and with his gain in flesh all his distressing symptoms vanished. After he returned home he wrote me that many of his life-long friends did not recognise him. He now weighs about 185 lbs., is well and at work in Oakland, Cal

The first week or two while the patient is getting adjusted to this systematic diet often requires much attention on the part of the physician and frequently much persistent urging and encouraging the patient, so as to secure their continued coöperation. In closing I will say, it is most uncommon to find a patient who needs milk, who cannot take it in the quantity required by the system, provided a sufficient quantity of water also is taken.

I have rarely found any better results from peptonised milk, or milk with lime water added, than from plain milk, if required, given hot, and, if the tendency to the formation of large curds exists, diluted with one-half or one-third its volume of water. It also happens that when a small quantity of milk disagrees with the patient a large quantity will often agree, unless it is contraindicated by the condition of the patient's nutrition, in which case more water should be given. Beware also of the patient who says, "I cannot take so much at first, but I will take a little, and work up gradually to the amount you direct." If such a course is allowed failure will result in nearly every case.

If a certain diet is prescribed it should be done with the same definiteness with which we prescribe our medicines and the same exact obedience should be expected of the patient as in regard to our medical prescriptions.

